# Exploring the evolutionary characteristics between cultivated tea and its wild relatives using complete chloroplast genomes

**DOI:** 10.1186/s12862-021-01800-1

**Published:** 2021-04-30

**Authors:** Jiao Peng, Yunlin Zhao, Meng Dong, Shiquan Liu, Zhiyuan Hu, Xiaofen Zhong, Zhenggang Xu

**Affiliations:** 1 Hunan Research Center of Engineering Technology for Utilization of Environmental and Resources Plant, Central South University of Forestry and Technology, Changsha, 410004 Hunan People’s Republic of China; 2 Hunan Provincial Key Lab of Dark Tea and Jin-Hua, Hunan City University, Yiyang, 413000 Hunan People’s Republic of China; 3 Key Laboratory of National Forestry and Grassland Administration on Management of Western Forest Bio-Disaster, College of Forestry, Northwest A & F University, Yangling, 712100 Shaanxi People’s Republic of China

**Keywords:** Chloroplast genome, Cultivated tea, Evolution, *ycf1*, *Camellia*

## Abstract

**Background:**

Cultivated tea is one of the most important economic and ecological trees distributed worldwide. Cultivated tea suffer from long-term targeted selection of traits and overexploitation of habitats by human beings, which may have changed its genetic structure. The chloroplast is an organelle with a conserved cyclic genomic structure, and it can help us better understand the evolutionary relationship of *Camellia* plants.

**Results:**

We conducted comparative and evolutionary analyses on cultivated tea and wild tea, and we detected the evolutionary characteristics of cultivated tea. The chloroplast genome sizes of cultivated tea were slightly different, ranging from 157,025 to 157,100 bp. In addition, the cultivated species were more conserved than the wild species, in terms of the genome length, gene number, gene arrangement and GC content. However, comparing *Camellia sinensis* var. sinensis and *Camellia sinensis* var. assamica with their cultivars, the IR length variation was approximately 20 bp and 30 bp, respectively. The nucleotide diversity of 14 sequences in cultivated tea was higher than that in wild tea. Detailed analysis on the genomic variation and evolution of *Camellia sinensis* var. sinensis cultivars revealed 67 single nucleotide polymorphisms (SNPs), 46 insertions/deletions (indels), and 16 protein coding genes with nucleotide substitutions, while *Camellia sinensis* var. assamica cultivars revealed 4 indels. In cultivated tea, the most variable gene was *ycf1*. The largest number of nucleotide substitutions, five amino acids exhibited site-specific selection, and a 9 bp sequence insertion were found in the *Camellia sinensis* var. sinensis cultivars. In addition, phylogenetic relationship in the *ycf1* tree suggested that the *ycf1* gene has diverged in cultivated tea. Because *C. sinensis* var. sinensis and its cultivated species were not tightly clustered.

**Conclusions:**

The cultivated species were more conserved than the wild species in terms of architecture and linear sequence order. The variation of the chloroplast genome in cultivated tea was mainly manifested in the nucleotide polymorphisms and sequence insertions. These results provided evidence regarding the influence of human activities on tea.

**Supplementary Information:**

The online version contains supplementary material available at 10.1186/s12862-021-01800-1.

## Background

From ancient times, numerous plant species have been taken from their habitats and introduced into cultivation—that is, into various human-made systems [1]. The cultivation process has played an important role in human history and cultivated environments often present strong ecological contrasts with wild environments [2]. Wild species are exposed to natural selection that operates to promote survival under abiotic and biotic stresses, while cultivated species are subjected to artificial selection that emphasizes a steady supply, improved quality and increased yield. The criteria for fitness are expected to change dramatically under both regimes. Therefore, alterations in vegetation phenology, growth and reproductive traits occur because the plants are subjected to different levels of stress and distinctive selection pressures [3]. Pot experiments showed there were significant differences in the flowering and pod set between wild and cultivated types of soybean [4]. In addition, the compounds and microstructures have been surveyed for many horticultural plants [5]. The inadequate genetic information prevents us from fully understanding the spreading process of cultivated plants. We need to compare the genetic differences between cultivated species and wild species in order to use these species more effectively.

*Camellia*, containing approximately 280 species, is a genus with high economic, ecological and phylogenetic values in the family Theaceae [6, 7]. *Camellia* are native to Asia and have been cultivated for more than 1300 years [8]. Because their variety of uses, the cultivated species are now found all over the world [9, 10]. *Camellia* species can provide many valuable products, including making tea with the young leaves and extracting edible oil from the seeds. Moreover, most *Camellia* species are also of great ornamental value [11]. The genus *Camellia* is composed of more than 110 taxa [12], of which *Camellia sinensis* (L.) O. Kuntze is the most important source of the beverage tea. Cultivated tea plant varieties mainly belong to two major groups: *Camellia sinensis* var. sinensis (CSS; Chinese type) and *Camellia sinensis* var. assamica (CSA; Assam type) [13]. Due to long-term cultivation and manual selection, *C. sinensis* formed many local varieties, such as *Camellia sinensis* var. sinensis cv. Anhua (CSSA), *Camellia sinensis* var. sinensis cv. Longjing43 (CSSL), *Camellia sinensis* var. assamica cv. Yunkang10 (CSAY) and so on. Wild tea plants are important genetic diversity resources that can provide new traits for improved yield, disease resistance and tolerance to different environmental conditions. For example, the leaves of CSSA, well known for its specific area, are the main sources of dark tea [14]. The quality of dark tea products is related to the abundant cultivars, germplasm resources and geographical conditions [15].

The chloroplast (cp) genome is often used to analyze the evolutionary process and the phylogenetic status because of its high degree of conservation and relatively compact gene alignment. Moreover, cp genome sequences are useful in the identification of closely related, breeding-compatible plant species [16]. Although the cp genome is very useful, there are still a limited number of full cp genomes available from *Camellia* species so far [7, 14, 17–21].

It has been proven that human interference has effects on the genetic structure, leaf nutrients and pollen morphology of *Camellia* [22–24]. For example, due to human overexploitation of habitats and long-term targeted selection of traits, the genetic diversity of *Camellia* germplasm resources has been significantly reduced [25]. Thus, it remains unclear what impact the artificially selected cultivated *Camellia* has had on the evolutionary mechanism of the cp genome.

Current research often ignores material differences between cultivated and wild species. After sequencing the complete chloroplast genome of CSSA (MH042531), we wanted to explore evolutionary characteristics between cultivated tea and its wild relatives [14]. To assess the variations in the chloroplast genome in wild and cultivated species of *Camellia*, and to detect the evolutionary characteristics of cultivated tea, we selected earlier published *Camellia* chloroplast genomes and conducted comparative and evolutionary analysis. This can help us to better understand the structure of the *Camellia* chloroplast genomes and the phylogenetic relationships among species, and provide more information about the influence of human activities on tea. We believe that this research will encourage more researchers to pay attention to tea resources.

## Results

### Chloroplast genome features of cultivated tea

**Fig. 1:**
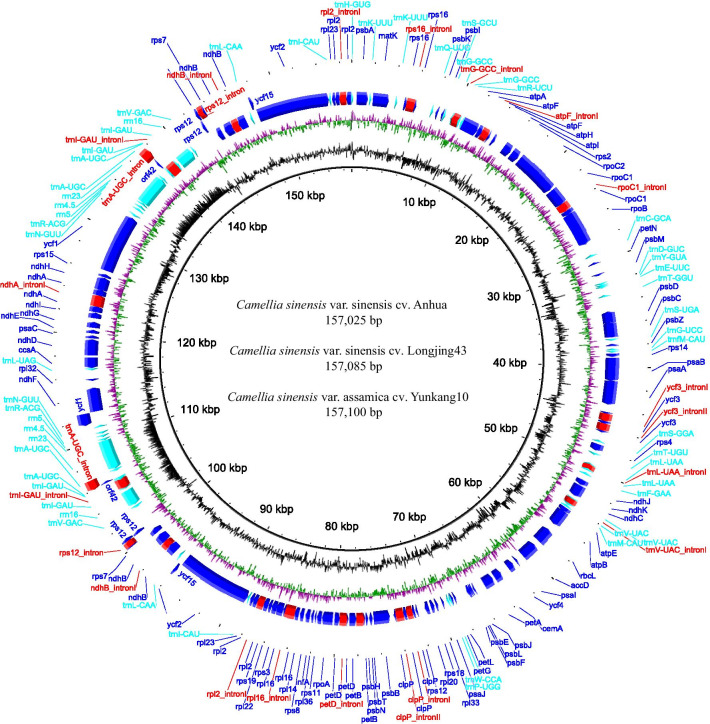
Gene map of the complete chloroplast genome of cultivated tea. The inner circle corresponds to the GC content, and the next circle corresponds to the GC skew. The next three circles correspond to the genes. Genes with clockwise arrows represent reverse strands, while genes with counterclockwise arrows represent forward strands. Blue, red and aqua colors of the blocks represent protein-coding genes, introns and RNA, respectively. The third circle corresponds to the shared genes among three cultivated tea. The fourth circle corresponds to the unique genes of *Camellia sinensis* var. sinensis Anhua and *Camellia sinensis* var. sinensis Longjing43. The fifth circle corresponds to the unique genes of *Camellia sinensis* var. assamica cv. Yunkang10

The lengths of the whole genomes of cultivated tea (CSSA, CSSL and CSAY) were slightly different, ranging from 157,025 to 157,100 bp. However, compared with CSSA and CSSL, the genome of CSAY was different. Both CSSA and CSSL contained 81 unique CDS genes, 30 tRNA, 4 rRNA and 3 pseudogenes (*ψycf1*, *ψycf2* and *ψycf15*). Among them, *atpF*, *ndhA*, *ndhB*, *petB*, *petD*, *rpl2*, *rpl16*, *rpoC1*, *rps16*, *trnG-GCC*, *trnI-GAU*, *trnL-UAA*, and *trnV-UAC* contained a single intron, while *clpP* and *ycf3* contained two introns. However, in CSAY, *orf42* and *ycf15* were lost, and *rps12* and *trnA-UGC* had an inserted intron sequence (Fig. 1).

### Comparison of chloroplast genomes between cultivated tea and wild tea

In our study, first, we compared CSS with its two cultivated species (CSSA and CSSL). These species were defined as the Chinese cultivated type. Then, we compared CSA with its one cultivated species (CSAY). These species were defined as the Assam cultivated type. Finally, we compared CSS, CSA and 12 wild but related species: *Camellia azalea* (CAZ), *Camellia crapnelliana* (CCR), *Camellia cuspidate* (CCU), *Camellia grandibracteata* (CGR), *Camellia impressinervis* (CIM), *Camellia petelotii* (CPE), *Camellia pitardii* (CPI), *Camellia pubicosta* (CPU), *Camellia reticulata* (CRE), *Camellia sinensis var. pubilimba* (CSP), *Camellia taliensis* (CTA) and *Camellia yunnanensis* (CYU). These species were defined as the wild type (Tables 1 and 2).

**Table 1:** Chloroplast genomic features of seventeen *Camellia* species

Species	CSS	CSSA	CSSL	CSA	CSAY	CSP	CGR	CTA	CIM	CPU	CAZ	CPI	CRE	CCR	CCU	CPE	CYU
Genome(bp)	157,117	157,025	157,085	157,028	157,100	157,086	157,127	156,974	156,892	157,076	157,039	156,585	156,971	156,997	156,618	157,121	156,592
CDS (bp)	80,542	80,620	80,650	79,093	79,092	80,622	80,656	79,577	79,655	80,665	80,629	79,619	76,224	79,649	79,643	80,650	79,655
Introns (bp)	15,192	15,196	15,198	17,902	17,902	15,210	15,205	16,947	16,897	15,198	15,195	16,937	15,182	16,239	16,917	15,196	16,935
IGS (bp)	49,535	49,361	49,389	48,200	48,268	49,405	49,418	48,591	48,481	49,365	49,367	48,171	53,717	49,321	48,199	49,427	48,143
tRNA (bp)	2802	2802	2802	2789	2790	2802	2802	2813	2813	2802	2802	2812	2802	2742	2813	2802	2813
rRNA (bp)	9046	9046	9046	9044	9048	9047	9046	9046	9046	9046	9046	9046	9046	9046	9046	9046	9046
Genes	115	115	115	113	113	115	115	115	115	115	115	115	115	114	115	115	115
CDS genes	81	81	81	79	79	81	81	81	81	81	81	81	81	81	81	81	81
tRNA genes	30	30	30	30	30	30	30	30	30	30	30	30	30	29	30	30	30
Introns	18	18	18	22	22	18	18	21	21	18	18	21	18	20	21	18	21
Genome GC	37.3	37.3	37.29	37.3	37.29	37.32	37.29	37.32	37.33	37.3	37.3	37.34	37.31	37.3	37.31	37.29	37.33
CDS GC	37.58	37.57	37.56	37.47	37.47	37.58	37.56	37.57	37.54	37.57	37.56	37.56	37.54	37.54	37.56	37.56	37.54
Introns GC	36.41	36.38	36.38	37.91	37.91	36.42	36.39	37.25	37.28	36.42	36.41	37.22	36.4	37.54	37.25	36.41	37.25
IGS GC	32.93	32.94	32.94	32.48	32.46	32.97	32.94	32.68	32.72	32.93	32.95	32.71	33.39	32.64	32.63	32.92	32.68
tRNA GC	52.86	52.86	52.86	52.99	52.97	52.86	52.86	52.86	52.9	52.89	52.86	52.92	52.86	52.88	52.9	52.86	52.9
rRNA GC	55.39	55.41	55.41	55.40	55.39	55.41	55.41	55.38	55.41	55.42	55.39	55.36	55.34	55.41	55.38	55.39	55.41
Gene losses				*orf42,* *ycf1,* *ycf15*	*orf42,* *ycf1,* *ycf15*			*orf42,* *ycf1*	*orf42,* *ycf1*			*orf42,* *ycf1*		*orf42,* *ycf1,* *trnG*	*orf42,* *ycf1*		*orf42,* *ycf1*
Intron losses	*rps12*	*rps12*	*rps12*			*rps12*	*rps12*			*rps12*	*rps12*		*rps12*			*rps12*	

CSS: *Camellia sinensis* var. sinensis, CSSA: *Camellia sinensis* var. sinensis cv. Anhua, CSSL: *Camellia sinensis* var. sinensis cv. Longjing43, CSA: *Camellia sinensis* var. assamica, CSAY: *Camellia sinensis var.* assamica cv. Yunkang10, CSP: *Camellia sinensis* var. pubilimba, CSP; *Camellia grandibracteata,* CTA: *Camellia taliensis*, CIM: *Camellia impressinervis*, CPU: *Camellia pubicosta*, CAZ: *Camellia azalea*, CPI: *Camellia pitardii*, CRE: *Camellia reticulata*, CCR: *Camellia crapnelliana*, CCU: *Camellia cuspidate*, CPE: *Camellia petelotii*, CYU: *Camellia yunnanensis*

**Table 2:** Information regarding the complete chloroplast genomes of the research species

Species	Accession number	Subgenus^1^	Section^1^	Types	Sample location	Location	References
*Camellia sinensis* var. sinensis	KJ806281	*Thea*	*Thea*	Wild	Yunnan Academy of Agricultural Science	Yunnan, China	[66]
*Camellia sinensis* var. sinensis cv. Anhua	MH042531	*Thea*	*Thea*	Cultivar	Hunan City University	Hunan, China	[14]
*Camellia sinensis* var. sinensis cv. Longjing43	KF562708	*Thea*	*Thea*	Cultivar	Huajiachi campus of Zhejiang University	Zhejiang, China	[17]
*Camellia sinensis* var. assamica	MH394410	*Thea*	*Thea*	Wild	Kunming Institute of Botany, Kunming	Yunnan, China	[21]
*Camellia sinensis var.* assamica cv. Yunkang10	MH019307	*Thea*	*Thea*	Cultivar	Menghai County	Yunnan, China	[67]
*Camellia sinensis* var. pubilimba	KJ806280	*Thea*	*Thea*	Wild	Yunnan Academy of Agricultural Science	Yunnan, China	[66]
*Camellia grandibracteata*	NC024659	*Thea*	*Thea*	Wild	Yunnan Academy of Agricultural Science	Yunnan, China	[66]
*Camellia taliensis*	NC022264	*Thea*	*Thea*	Wild	Kunming Institute of Botany	Yunnan, China	[7]
*Camellia impressinervis*	NC022461	*Thea*	*Archecamellia*	Wild	Kunming Institute of Botany	Yunnan, China	[7]
*Camellia pubicosta*	NC024662	*Thea*	*Corallina*	Wild	International Camellia Species Garden	Zhejiang, China	[66]
*Camellia azalea*	NC035574	*Camellia*	*Camellia*	Wild	Yangchun County	Guangdong, China	[19]
*Camellia pitardii*	NC022462	*Camellia*	*Camellia*	Wild	Kunming Institute of Botany	Yunnan, China	[7]
*Camellia reticulata*	NC024663	*Camellia*	*Camellia*	Wild	Kunming Institute of Botany	Yunnan, China	[66]
*Camellia crapnelliana*	NC024541	*Camellia*	*Heterogenea*	Wild	Kunming Botanical Garden	Yunnan, China	[20]
*Camellia cuspidata*	NC022459	*Thea*	*Theopsis*	Wild	Kunming Institute of Botany	Yunnan, China	[7]
*Camellia petelotii*	NC024661	*Thea*	*Archecamellia*	Wild	International Camellia Species Garden	Zhejiang, China	[66]
*Camellia yunnanensis*	NC022463	*Camellia*	*Heterogenea*	Wild	Kunming Institute of Botany	Yunnan, China	[7]

^1 ^The taxonomic classification of *Camellia* is based on Ming’s research [47]

### Chloroplast genomic similarity

In the Chinese cultivated type, the average length across the cultivated species was 62 bp smaller than CSS. In the Assam cultivated type, the genome length of CSAY was 72 bp larger than CSA. In the wild type, the average length of the wild species was 156,923 bp, which was 194 bp and 105 bp variation compared with CSS and CSA, respectively. This showed that there was less length variation when comparing cultivated species with wild species (Table 1). Similarly, the number of genes and the GC content of cultivated species were more stable than that of wild species. After comparing the genes and introns insertion or deletion among the Chinese cultivated type, Assam cultivated type and wild type, we found that introns of the *rps12* gene were deleted in CSS and its two cultivated species. The *orf42*, *ycf1* and *ycf15* genes were deleted in CSA and CSAY. However, these events occurred randomly in wild species. The differences in the GC content of the CDS, intron and IGS in the Chinese cultivated type and Assam cultivated type were approximately 0.01–0.03%, and 0–0.02%, respectively, but we found that the differences of the CDS, intron and IGS in the wild type were 0.02–1.05%.

**Fig. 2:**
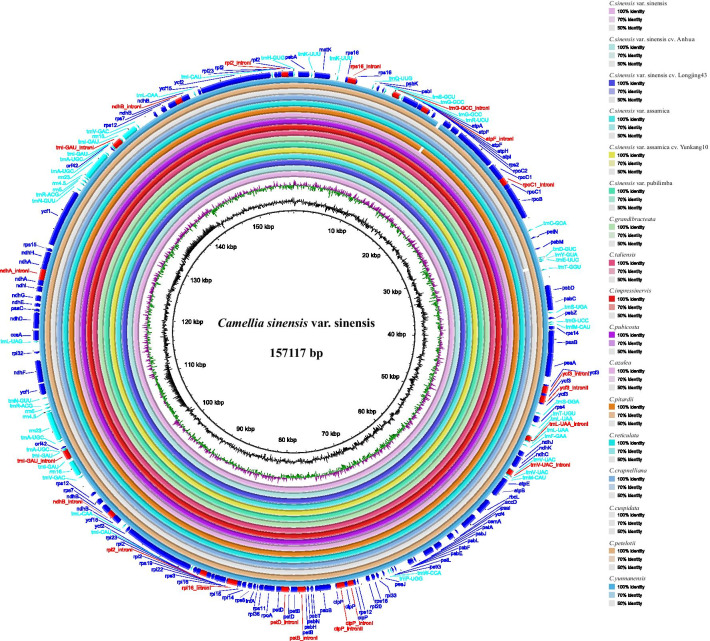
The sequence identity of seventeen *Camellia* species. The inner circle is the reference genome. Next circles represent the sequence identity between *C.sinensis* var. sinensis and sixteen other species. The outermost circle corresponds to the protein-coding genes and intergenic spacer regions. Genes with clockwise arrows represent reverse strands, while genes with counterclockwise arrows represent forward strands

**Fig. 3:**
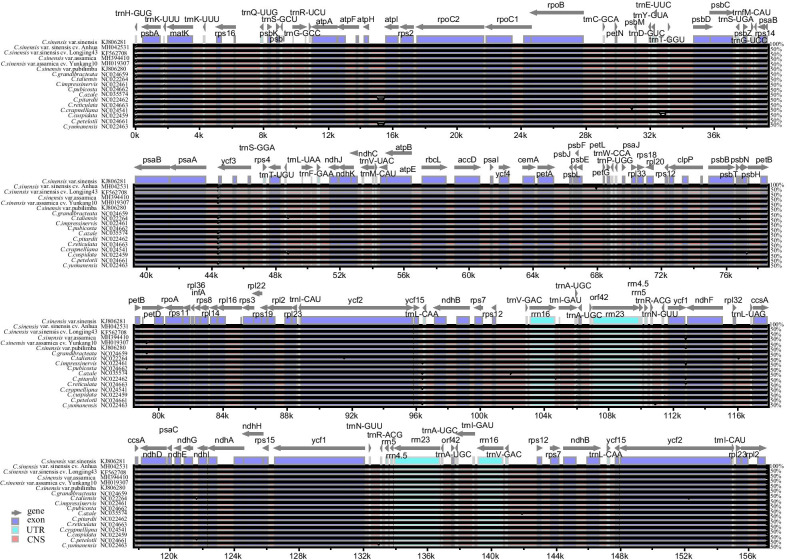
Alignment visualization of the seventeen *Camellia* chloroplast genome sequences using *C.sinensis* var. sinensis as a reference. The vertical scale indicates the percentage of identity, ranging from 50 to 100%. Arrows indicate the annotated genes and their transcriptional direction. The different colored boxes correspond to exons, tRNA or rRNA, and noncoding sequences (CNSs)

mVISTA and Blast Ring Image Generator (BRIG) were used to compare the genomic sequence identity. Comparing CSS and CSA with their cultivated types, the regions with relatively low identity were *psaA*_*ycf3*, *petL*_*petG* and *ycf1*_*ndhF*. Comparing CSS and CSA with other wild types, the regions with relatively low identity were *atpH*_*atpI*, *trnE-UCC*_*trnT-GGU*, *psaA*_*ycf3*, *ycf15*_*trnL-CAA*, *ycf1*_*ndhF* and *ndhG*_*ndhI* (Figs. 2 and 3). In conclusion, at the genomic level, the cultivated species were more conserved than the wild species.

### The expansion and contraction of IR regions

**Fig. 4:**
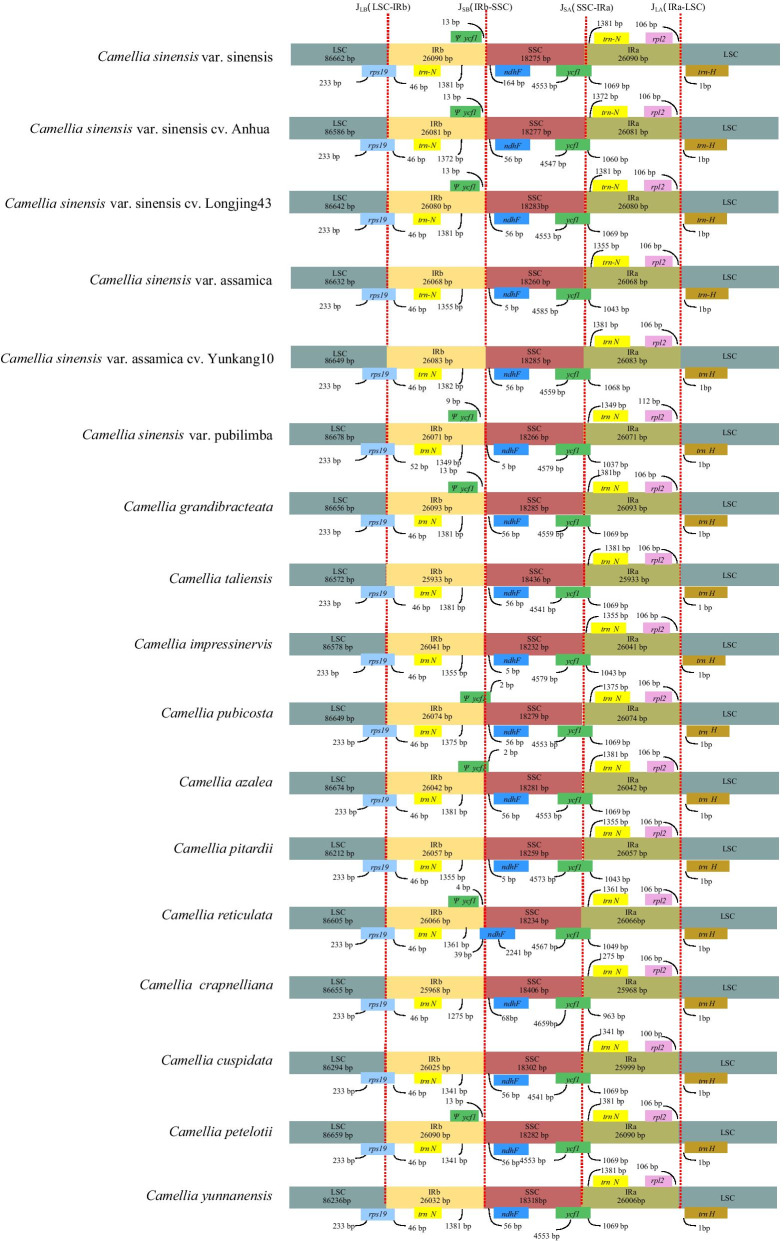
Comparison of IR boundary regions among the 17 *Camellia* chloroplast genomes, using *C. sinensis* var. sinensis as the reference. Boxes above or below the line are forward strands and reverse strands, respectively

The locations of inverted repeat (IR) regions were extracted via a self-BLASTN search, and the characteristics of the IR/Large single copy region (LSC) and IR/Small single copy region (SSC) boundary regions were analyzed. The IRs boundary regions of the 17 complete *Camellia* cp genomes were compared, showing slight differences in junction positions (Fig. 4). In order to detect possible IR border polymorphisms, first of all, we compared the four IR boundaries of the Chinese cultivated type. No difference was found at the LSC/IRb or IRa/LSC border; meanwhile, only minor differences were discovered at the IRb/SSC and SSC/IRa borders. Next, we compared the four IR boundaries of the Assam cultivated type, and the results were similar. Then, we compared the cp genome boundaries of the wild type. The *rps19* gene at the LSC/IRb boundary expanded 52 bp from the LSC region to the IRb side in CPU, while it stopped at 46 bp from the LSC region in the rest of the species. On the other side of the IRa/LSC boundary, the lengths of the spacers between the IRa/LSC junction and the *rpl2* gene (in IRa) were 112 bp for CPU, while those of the rest of the species were all 106 bp. Consistently, in all of the compared cp genomes, the *ycf1* gene spanned the SSC/IRa region and the length of *ycf1* ranged from 963 to 1069 bp in IRa. Remarkably, most species have an *ycf1* pseudogene at the IRa/LSC junction, while this was not observed in CSA, CTA, CIM, CPI, CCR, CCU, or CYU. Similar to most plants, the *ndhF* gene involved in photosynthesis was located in the SSC region. However, the *ndhF* gene was located at the IRb/SSC boundary of CRE, and there was a 35 bp overlap between *ndhF* gene and *ψycf1*gene.

### Nucleotide diversity

**Fig. 5:**
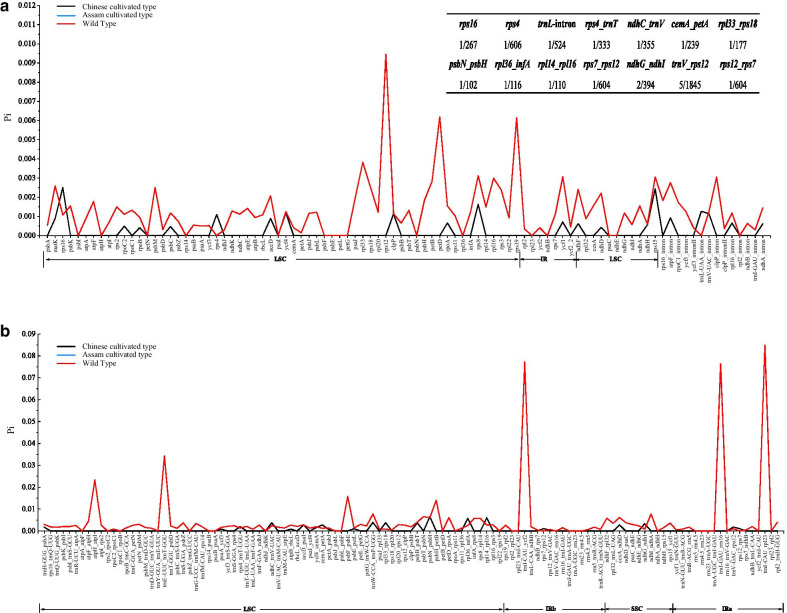
Comparative analysis of nucleotide variability (Pi) values among Chinese cultivated type, Assam cultivated type and wild type. X-axis: the names of protein-coding genes, introns or intergenic regions, Y-axis: nucleotide diversity of each window

Comparisons based on the nucleotide diversity (Pi) values of the Chinese cultivated type, Assam cultivated type, and wild type were presented, including the intergeneric regions (IGS), protein-coding genes and introns (Additional file 1: Table S1, Fig. 5). In our study, the average Pi values for the genes, introns and IGS in wild type were approximately 6.6, 3.5 and 9.1 times that of the Chinese cultivated type. In addition, the Pi values for all regions in the Assam cultivated type were 0. Comparing Chinese cultivated type with wild type, the Pi values of most genes, introns and IGS in the wild species were higher than those of in the cultivated species. For example, *rps12*, *petD*, *rps19*, *trnI-CAU_rpl23*, *trnI-CAU_ycf2*, *trnI-GAU_rrn16*, *clpP*_intron, *rps16*_intron, and *atpF*_intron were highly variable in the wild species, but they were not variable in the three cultivated species. For the photosynthetic genes, except for *ndhD, ndhF, ndhH* and *psbC*, the Pi values of the photosynthetic genes of three cultivated tea were 0. The Pi values of these genes were smaller than that of the wild species. These results indicate that these genes and noncoding regions were more conserved among the cultivated species than among the wild species.

Furthermore, although the average Pi values of the cultivated species were lower, we still found that the Pi values of *rps16*, *rps4*, *trnL-UAA*_intron, *rps4*_*trnT-UGU*, *ndhC*_*trnV-UAC*, *cemA*_*petA*, *rpl33*_*rps18*, *psbN*_*psbH*, *rpl36*_*infA*, *rpl14*_*rpl16*, *rps7*_*rps12*, *ndhG*_*ndhI*, *trnV-GAC*_*rps12*, and *rps12*_*rps7* in the Chinese cultivated type were higher than those in wild species, and these difference sequences were mainly located in the LSC region (Fig. 5).

### Phylogenetic analysis of cultivated tea and wild tea

**Fig. 6:**
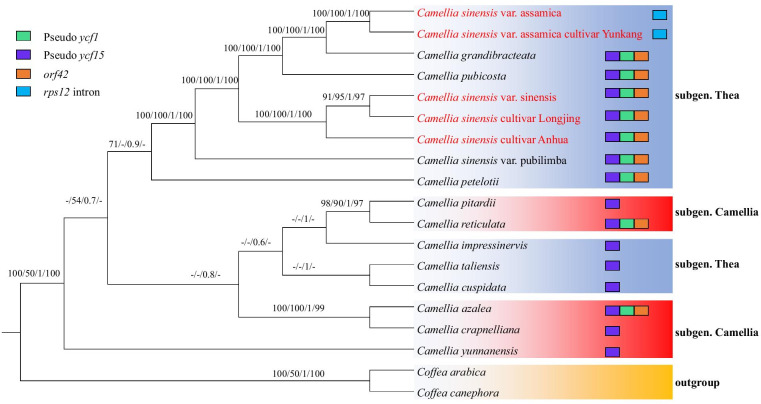
The phylogenetic tree of *Camellia* species based on the complete cp genomes (complete cp-Tree). *Coffea canephora* and *Coffea arabica* were selected as the outgroup. Tree were constructed by neighbor-joining (NJ), maximum parsimony (MP), Bayesian inference (BI) and maximum likelihood (ML) with bootstrap values or posterior probabilities above the branches, respectively. Bootstrap values less than 50% are represented by "-". As indicated in the legend at the top left, the unique genes and introns of each species were plotted onto branches using colored squares

**Fig.7:**
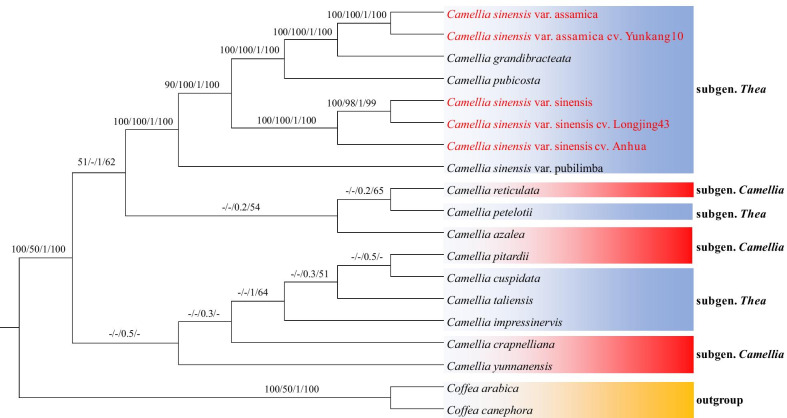
The phylogenetic tree of *Camellia* species based on the all shared coding protein genes among all species (SCDS-Tree). *Coffea canephora* and *Coffea arabica* were selected as the outgroup. Tree were constructed by neighbor-joining (NJ), maximum parsimony (MP), Bayesian inference (BI) and maximum likelihood (ML) with bootstrap values or posterior probabilities above the branches, respectively. The bootstrap values less than 50% are represented by "-"

**Fig. 8:**
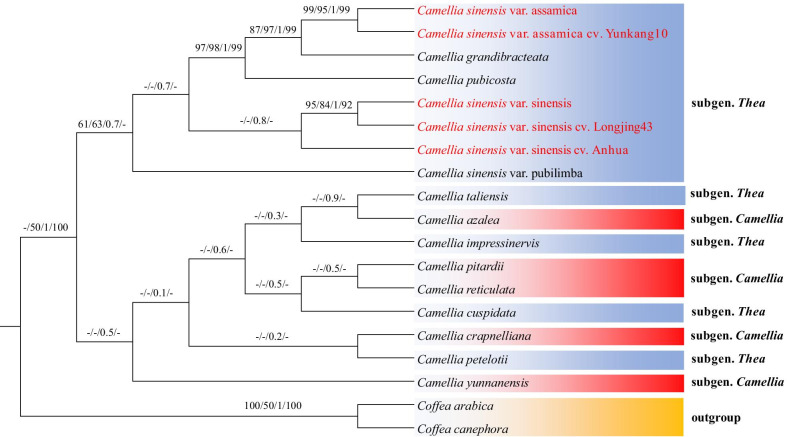
The phylogenetic tree of *Camellia* species based on the *ycf1* gene (*ycf1*-Tree). *Coffea canephora* and *Coffea arabica* were selected as the outgroup. Tree were constructed by neighbor-joining (NJ), maximum parsimony (MP), Bayesian inference (BI) and maximum likelihood (ML) with bootstrap values or posterior probabilities above the branches, respectively. The bootstrap values less than 50% are represented by "-"

We constructed three phylogenetic trees of cultivated and wild tea, namely, the complete cp genomic tree (complete cp-Tree), all shared protein coding genes among all species tree (SCDS-Tree) and the *ycf1* gene tree (*ycf1*-Tree) (Figs. 6, 7 and 8). All phylogenetic trees supported the hypothesis that the *Thea* subgenus could be divided into two clades: clade I, including CSS, CSSL, CSSA, CSA, CSAY, CGR, CPU and CSP, and clade II, including CPE CIM, CTA and CCU. Clade I was strongly supported, because the posterior probabilities or bootstrap values obtained by neighbor-joining (NJ), maximum parsimony (MP), Bayesian inference (BI) and maximum likelihood (ML) were very high for each lineage. These results suggested that the seven species in clade I were closely related. All phylogenetic trees proved that CSS was the closest relative to CSSA and CSSL, and CSA was the closest relative to CSAY. In particular, in the *ycf1*-Tree, the posterior probabilities or bootstrap values of these species were lower than those of the complete cp-Tree and the SCDS-Tree. The value of CSSA was less than 50%. These results suggested that the *ycf1* gene has diverged in cultivated tea.

In addition, we found conflict among the three trees (Figs. 6, 7 and 8). The topological structures consisting of the *Camellia* subgenus (CPI, CRE, CAZ, CCR, and CYU) and the *Thea* subgenus (CPE, CIM, CTA and CCU) were poorly supported by the complete cp-Tree, SCDS-Tree and *ycf1*-Tree, because most bootstrap values or posterior probabilities were less than 50% for each lineage. These results may be caused by unbalanced sampling.

The cp-Tree showed some structural variations among the *Camellia* cp genomes (Fig. 6). The clade, which was made up of CSS, CSSL, CSSA, CSA, CSAY, CGR, CPU, CSP and CPE, was characterized by the *rps12* intron deletion, the *ψycf1* gene, and the *ψycf15* gene (except for CSA and CSAY)*.* The other species, except for CRE and CAZ, had lost the *ψycf1* gene and the *orf42* gene.

### Chloroplast genome variation and evolution in cultivated tea

To explain the changes in the cp genome structure of the cultivated tea group, we detected single nucleotide polymorphism (SNP) and insertion/deletion (indel) in the cp genome of cultivated tea. In the Chinese cultivated type, after comparing the whole cp genome of three species, 67 SNPs and 46 indels were found. The LSC, IRb, SSC and IRa regions contained 43, 3, 13, and 8 SNPs and 37, 2, 5, and 2 indels, respectively (Additional file 2: Table S2). Most of the SNPs and indels were located in the noncoding region (IGS and intron). There were 39 SNPs and 41 indels in this region, while 28 SNPs and 5 indels were found in the protein coding region. The two *ycf1* genes, which are located at the junction of SSC and IRa, contained the most SNPs and indels, 6 and 2, respectively. For the photosynthetic genes, *psbC*, *ndhD*, *ndhF* and *ndhH* presented SNP variations, while the *psbI* gene presented indel variation. For the 14 sequences with higher Pi values in cultivated species than in wild species, *trnV-GAC_rps12* and *ndhG_ndhI* contained the most abundant SNPs, with 5 and 2 respectively (Fig. 5). In the Assam cultivated type, after comparing the whole cp genome of two species, 4 indels were found, but no SNPs. All indels were located in the IGS region. In particular, a long sequence (77 bp) was inserted into the IRb/SSC boundary region (Additional file 3: Table S3).

**Fig. 9:**
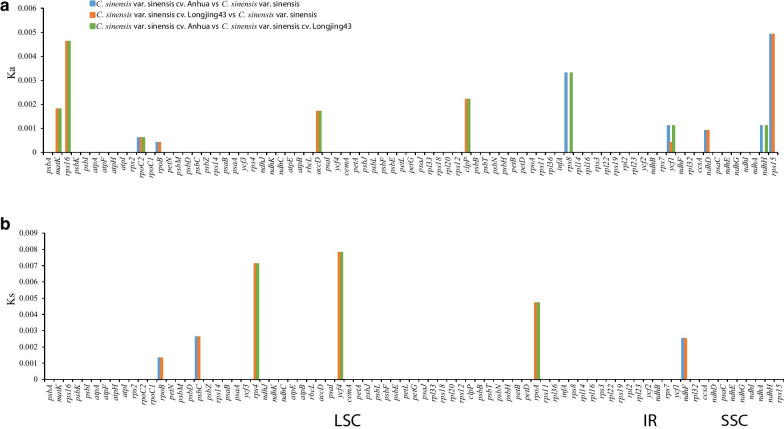
Nonsynonymous nucleotide substitution (Ka) and synonymous nucleotide substitution (Ks) of homologous protein-coding genes from *C. sinensis* var. sinensis, *C. sinensis* var. sinensis cv. Longjing43 and *C. sinensis* var. sinensis cv. Anhua

To have a clear view of the evolution of cultivated species, we used their 80 shared protein coding genes to calculate their nonsynonymous nucleotide substitution (Ka) rates, synonymous nucleotide substitution (Ks) rates and Ka/Ks ratio. First, we compared CSS and its cultivated species. The results showed that only 16 protein coding genes had synonymous or nonsynonymous mutations (Fig. 9, Additional file 4: Table S4). Among them, there were nonsynonymous mutations in *matK*, *rps16*, *rpoC2*, *rpoB*, *accD*, *clpP*, *rps8*, *ycf1*, *ndhD*, *ndhH* and *rps15*. The genes with the highest rate of nonsynonymous mutations were *rps16, rps8* and *rps15*. There were synonymous mutations in *rpoB, psbC, rps4, ycf4, rpoA* and *ndhF*. The highest mutation rates were *rps4, ycf4* and *rpoA*. Of the 80 genes, 79 had a Ka / Ks value of 0, and only *rpoB*, had a Ka/Ks value of 0.3004 < 0.5, suggesting very strong purifying selective pressure. Then, we compared CSA and its cultivated species. However, no protein coding genes had synonymous or nonsynonymous mutations, suggesting very strong purifying selective pressure (Additional file 5: Table S5).

The site specific selection events of 16 genes with synonymous or non-synonymous mutations were analyzed by Bayesian Empirical Bayes (BEB), and we found that some amino acid sites of *ycf1* and *rps15* exhibited site-specific selection (Additional file 6: Table S6). In *ycf1*, there were six sites under positive selection, and in *rps15*, there was one site under positive selection. For example, in the *rps15* gene, the codon ACC (threonine) of CSS was mutated to AAC (asparagine) in two cultivated species.

## Discussion

Understanding the genetic variation between cultivated and wild species is crucial for introducing interesting traits from wild species into cultivars [26]. Organelle genome sequencing has proven to be an effective way to resolve phylogenetic relationships among closely related species [27, 28]. Here, we constructed and compared the complete cpDNA genome sequences of three cultivars and fourteen wild species of *Camellia*. At the genomic level, cultivated species were more conserved than wild species, in terms of both architecture and linear sequence order (the length, genes number, genes arrangement, and GC content) (Table 2, Figs. 2 and 3). For other land plant species, such as peanuts, cherries and radishes, the cp genome size and structure, as well as the gene content and order, are highly conserved among the cultivated and wild species [29–31].

We found that the IR regions of cultivated tea had expanded or contracted. The IR length of the CSSA and CSSL was approximately 20 bp smaller than that of the CSS, accounting for 32% of the difference in the complete genome length. The IR length of the CSAY was approximately 30 bp larger than that of the CSA, accounting for 42% of the difference in the complete genome length (Fig. 4). In fact, the contraction and expansion of IRs is considered to be one of the important reasons for the cp genome length variation [32]. Further SNP and indel analysis showed that *ycf1* and *trnV-GAC*_*rps12* changed in the Chinese cultivated type, while *trnN-GUU*_*ndhF* and *rrn5*_*trnR-ACG* changed in the Assam cultivated type. In CSS and CSSL, a 9 bp sequence (TCCTTCTTC/GAAGAAGGA) was inserted into the *ycf1* gene (Additional file 2: Table S2). This is suggested that *ycf1* is one of the important reasons for the expansion or contraction of the IRs of the Chinese cultivated type. The same results were also found in Zheng’s study [33]. He analyzed the cp genome length variation in 272 species and found that *atpA*, *accD* and *ycf1* accounted for 13% of the difference in length. Therefore, *ycf1*, which is associated with plant survival, may play a key role in the cp genome size variations of cultivated tea. In CSAY, a 77 bp sequence was inserted into the *trnN-GUU_ndhF* region (IRb/SSC boundary region) (Additional file 3: Table S3). This is the main reason for the expansion or contraction of the IRs of the Assam cultivated type.

In addition to the variations in genome size, there were also some nucleotide mutations in the cultivated species. In this study, the nucleotide diversity of cultivated tea was lower than that of wild tea (Fig. 5), but the unbalanced sampling between the 14 wild tea and 3 cultivated tea may lead to nucleotide diversity difference of cpDNA fragments. The nucleotide diversity comparison of 358 cultivated rice and 54 wild rice also presented similar results [34]. Nevertheless, we found that the nucleotide diversity of 14 sequences in the Chinese cultivated tea was higher than that of wild tea (*rps16*, *rps4*, *trnL-UAA*_intron, *rps4*_*trnT-UGU*, *ndhC*_*trnV-UAC*, *cemA*_*petA*, *rpl33*_*rps18*, *psbN*_*psbH*, *rpl36*_*infA*, *rpl14*_*rpl16*, *rps7*_*rps12*, *ndhG*_*ndhI*, *trnV-GAC*_*rps12*, and *rps12*_*rps7*) (Fig. 5). These sequences suggested the variations in the cp genomes of cultivated tea, and they are potential molecular markers for distinguishing *Camellia* species and for the phylogenetic analysis of *Camellia*.

Previous studies have proven that human interference had effects on the genetic structure, leaf nutrients and pollen morphology of *Camellia*. Yan et al. analyzed the genetic relationship of five semi-wild tea which due to lack of human management for a long time were studied by using genome-wide SNP. They found that human interference will affect the genetic structure of tea. After the human interference stopped, the tea from five different geographical regions could be divided into three different groups because of the absence of free pollination [22]. Xiong et al. made comparative analyses of the nutrient content in the leaves of cultivated and wild *C. nitidissima*. They found that cultivated *C. nitidissima* had significantly higher contents of essential amino acids (26.05%) and total amino acids (33.27%) than wild *C. nitidissima* [23]*.* Shu et al. proved that there are obvious differences in pollen morphology and exine morphology between cultivated and wild species of *Camellia* [24]. Therefore, to explore specific evolutionary characteristics between cultivated tea and its wild relatives, we subsequently performed evolutionary research on cultivated tea.

First, to have a clear view of the cp genomic adaptive evolution of cultivated tea, we performed evolutionary analysis on the protein-coding sequences. The Ka/Ks ratio is very useful for measuring selective pressure at the protein level [35]. In the Chinese cultivated type, Ka/Ks value of 79 genes was 0, and only *rpoB* had a value of 0.3004. In addition, some amino acids of *ycf1* and *rps15* exhibited site-specific selection (Additional file 4: Tables S4 and Additional file 6: S6). *rpoB* is crucial for genetic information transmission, and it affects the transcription of DNA into RNA and the translation of RNA into protein. They were also found to be under selective pressure in beverage crops [13]. The *rps15* gene has a function in chloroplast ribosome subunits [35]. *ycf1*, encoding a component of the chloroplast’s inner envelope membrane protein translocon, is one of the largest plastid genes [13], and it is also essential for almost all plant lineages [36]. These positively selected genes may have played key roles in the adaptation of cultivated tea to various environments.

Generally, the deletion or insertion of amino acids in the encoded protein will affect the structure and function of this gene [37–39]. In the Chinese cultivated type, 16 protein coding genes had nucleotide substitutions, among which the *ycf1* gene had the largest number of nucleotide substitution. At the same time, in *ycf1*, five amino acid sites exhibited site-specific selection, and a 9 bp sequence insertion was found in CSSA (Additional file 4: Table S4 and Additional file 6: S6, Fig. 9).

*ycf1* has an open reading frame of unknown function, but some studies have inferred that *ycf1* is very important for plant survival [33, 40]. In tobacco, a chimeric gene conferring resistance to aminoglycoside antibiotics has been transferred into *ycf1* in the cp genome. Then, the plantlets were cultured in plant regeneration medium containing the antibiotic spectinomycin. After that, the maintenance of a fairly constant ratio of wild-type versus transformed genome copies was found. However, the wild-type genome was still present in all samples whereas the transplastomic fragments were missing from several samples after culturing in antibiotic-free medium. This experiment proved that *ycf1* encodes products that are essential for cell survival. *ycf1* is also an important molecular marker of plants [41, 42], because it has higher variability than other known cp molecular markers (such as the widely used *rbcL* and *matk* genes), for both the total number of parsimony informative characters and the percent variability.

Phylogenetic analysis of cultivated and wild tea showed that CSSA and CSSL were closely related to the CSS, and CSAY was closely related to CSA (Figs. 6 and 7), which supports the previous finding that most of the cultivated tea originated directly from CSS and CSA [43]. However, in the *ycf1*-Tree, the posterior probabilities or bootstrap values of the cultivated tea branch were lower than that of the complete cp-Tree and the SCDS-Tree, which suggested that the *ycf1* gene has diverged in cultivated tea (Figs. 6, 7 and 8). Similar results have been found in *Corylus* [44]. The *ycf1* gene of *Corylus chinensis* and *Corylus avellana* have a similar evolutionary history, which is different from that of *Corylus heterophylla*. This evolution of cultivated plants may be related to the utilization efficiency of photosynthesis. Photosystem biogenesis regulator 1 (PBR1), the RNA binding protein encoded by the nuclear genome, can improve the translation efficiency of *ycf1* in the *Arabidopsis thaliana* cp genome. Additionally, the symbiosis and stability maintenance of the three photosynthetic complexes are regulated [45]. However, at present, the effect of mutations in the single amino acid site and the insertion or deletion of the short sequence on the function of *ycf1* is still not clear, and cultivated tea may provide important materials for this kind of research.

In the phylogenetic trees, CSS, CSA, CGR and CPU formed a monophyletic clade with 100% bootstrap values. CSS, CSA and CGR were classified into the sect. *Thea*, but CPU was classified into the sect. *Corallina* (Table 2). This indicates that CPU and sect. *Thea* plants have close genetic relationship. It also supports the result of Huang’s research [18]. However, CTA belongs to sect. *Thea*, together with two species of sect. *Archecamellia* and one species of sect. *Theopsis* that were located in another clade, which indicates that the phylogenetic direction of CTA is different from that of the other sect. *Thea* species. CTA is often considered to be a wild relative of cultivated tea [43]. Both are monoecious, insect-pollinated and outcrossing species. However, there are differences in their morphological characters. For example, CTA has the features of 5-locule ovaries and large sepals and petals, whereas CSS has features of 3-locule ovaries and small sepals and petals [46, 47]. Based on the evidence of the chloroplast genome, we hypothesized that CTA and CSS have different genetic polymorphism. In this study, CIM and CPE were not clustered into the same branch. The taxonomy of CIM is controversial. CIM and CPE were classified into the sect. *Archecamellia* by Ming et al. [47], while Chang et al. [46] classified CIM into the sect. *Chrysantha*. Therefore, we infer that it is not acceptable to combine the sect. *Archecamellia* and the sect. *Chrysantha*. In the subgenus *Camellia*, CPI and CRE formed a clade, as did CAZ and CCR, and the bootstrap value was 97–100%. Among them, CPI, CRE and CAZ are all sect. *Camellia* plants, while CCR is classified into sect. *Heterogenea* [47] or sect. *Furfuracea* [46]. However, both morphological and molecular characteristics indicate that CCR is closely related to some plants in sect. *Camellia* [48].

## Conclusion

In this work, the complete cp genomes of three cultivated species and 14 wild species of *Camellia* were studied. Genomic variation and evolutionary processes were compared in these species. Genomic variation analyses showed that the cultivated species were more conserved than the wild species in terms of architecture and linear sequence order. In the Assam cultivated type, the variation in the chloroplast genome was mainly manifested by sequence insertion of IGS regions. In the Chinese cultivated type, the variation in the chloroplast genome was mainly manifested by the nucleotide polymorphism and sequence insertion of some sequences. These nucleotide polymorphisms also led to the mutation of amino acid sites in some genes, among which *ycf1* was the gene with the most mutation sites. In addition to amino acid mutations, there was a 9 bp base insertion in the *ycf1* gene. *ycf1* is believed to be a critical gene for plant survival, and it may influence photosynthesis and be related to plant adaptation. Evolutionary processes analyses showed that CSA and its cultivated species were tightly clustered, while CSS and its cultivated species were not tightly clustered. The evolutionary relationship between CSS and CSSL was closer than that with CSSA in the *ycf1*-Tree. However, at present, the effect of the mutation in the single amino acid site and insertion or deletion of the short sequence on the function of *ycf1* are still not clear, and cultivated tea may provide important materials for this kind of research.

## Methods

### Genomic materials collection of cultivated tea

The complete cp genome of CSSA has been presented and annotated in our previous study [14] with GenBank accession number MH042531. Meanwhile, we searched in the National Center for Biotechnology Information (NCBI) dataset to find the published cultivated tea’s complete cp genomes, and only CSSL and CSAY with accession numbers KF562708 and MH019307 have been published [17]. Gene map of the three cultivated tea was generated using BRIG [49].

### Comparative analysis between cultivated tea and wild tea

The Basic Local Alignment Search Tool (BLAST) was used to find closely related cp genomes of CSSA in NCBI. After the cp genome of *Camellia* was screened, 17 *Camellia* cp genomes with sampling information remained, including 3 cultivated species (CSSA, CSSL and CSAY) and 14 wild species (Table 2). Previous studies have shown that both CSSA and CSSL originated directly from CSS, while CSAY originated directly from CSA [43, 49]. Therefore, we used CSS and CSA as the reference sequence to study the genomic variations and evolution direction between cultivated tea and wild tea.

Three methods were used for comparative genomic analysis: (I) The comparison of the cp genomic sequence identity was based on the method of Li [50] using mVISTA in Shuffle-LAGAN mode and BRIG, respectively. (II) The comparison of the expansion and contraction of IR regions was presented. First, we annotated and extracted the IR boundary of the *Camellia* cp genomes by Plastid Genome Annotator (PGA) [51]. Then, the IR boundary regions were visualized by using Visio professional 2016. (III) Comparisons based on the Pi values of the Chinese cultivated type, Assam cultivated type, and wild type were performed according to the method of Njuguna [52]. First, we used annotation information to extract intergenic regions, protein coding genes and intron regions of 17 *Camellia* species in Tbtools v0.6666 [53]. After comparing these sequences, 211 loci shared among *Camellia* species were found, including 80 protein coding genes, 117 intergenic regions, and 14 intron regions. Each loci was divided into three datasets: (I) the sequences consisted of the Chinese cultivated type, (II) the sequences consisted of the Assam cultivated type; (III) the sequences consisted of wild type. Each sequence was aligned using clustal alignment with default settings in MEGA7.0 [54]. The Pi of these regions was calculated using DnaSP v6.10.04 [55] to show divergence at sequence level.

### Phylogenetic analysis of *Camellia*

Three datasets were used to construct the following phylogenetic trees of *Camellia*: (I) the complete cp genomes, (II) the all shared protein coding genes among all species (SCDS), and (III) *ycf1* gene sequences. First, all datasets were aligned using MAFFT v7.380 [56] under the FFT-NS-2 default setting. The alignments were used for phylogenetic analysis. After that, according to the method described by Xie et al. [57] and Zhang et al. [58], we used four methods to construct phylogenetic trees: NJ method, MP method, BI method and ML method. *Coffea canephora* and *Coffea arabica* were selected as the outgroup.

The NJ analysis was reconstructed via MEGA7.0 [54] under the default settings with 1000 bootstrap values. The MP analysis was performed in PAUP 4.0a167 [59] with heuristic searches with 1000 bootstrap replicates. The BI analysis was performed with Mrbayes 3.2.7 [60] under the best substitution models and parameters. The analysis parameters were set as four chains that were run simultaneously for 10,000,000 generations or until the average standard deviation of the split frequencies fell below 0.01. The best substitution models and parameters were computed by jmodeltest 2.1.7 [61]. The ML analysis was carried out in IQ-TREE [62] using the default settings, with 1000 bootstrap values for tree evaluation. The best substitution models were computed by IQ-TREE. All the best substitution models mentioned earlier were listed in Additional file 7: Table S7.

### Evolutionary analysis of cultivated tea

After alignment of the cultivated and wild species, the number and position of SNPs and indels in the genomes were presented in DnaSP v6.10.04 according to the Wu’s method [63].

The Ka and Ks rates as well as the Ka/Ks ratio in the homologous protein-coding genes were used to evaluate the adaptive evolution of the cultivated species. After aligning each gene using the ClustalW (Codons) program in MEGA7.0, the Ks, Ka and Ka/Ks values of each gene were determined according to Dong’s method [64] with the program from the PAML package [65]. For identification of site-specific selection, four models, M1 (neutral), M2 (selection), M7 (beta) and M8 (beta & ω), were used in codeml from the PAML package. The BEB was used to calculate the posterior probabilities for site classes. Only sites with posterior probabilities > 0.9 were selected.

## Supplementary Information

**Additional file 1: Table S1.** Comparative analysis of nucleotide variability (Pi) values among the Chinese cultivated type, Assam cultivated type and wild type

**Additional file 2: Table S2.** Single nucleotide polymorphism (SNP) and insertion/deletion (indel) information from comparisons among *C. sinensis* var. sinensis, *C. sinensis* var. sinensis cv. Longjing43 and *C. sinensis* var. sinensis cv. Anhua

**Additional file 3: Table S3.** Single nucleotide polymorphism (SNP) and insertion/deletion (indel) information from comparisons between *C. sinensis* var. assamica and *C. sinensis* var. sinensis assamica cv. Yunkang10

**Additional file 4: Table S4.** Nonsynonymous nucleotide substitution (Ka) and synonymous nucleotide substitution (Ks) rates, as well as the Ka/Ks ratio of homologous protein-coding genes from *C. sinensis* var. sinensis, *C. sinensis* var. sinensis cv. Longjing43 and *C. sinensis* var. sinensis cv. Anhua

**Additional file 5: Table S5.** Nonsynonymous nucleotide substitution (Ka) and synonymous nucleotide substitution (Ks) rates, as well as the Ka/Ks ratio of homologous protein-coding genes from *C. sinensis* var. assamica and *C. sinensis* var. sinensis assamica cv. Yunkang10

**Additional file 6: Table S6.** Positive selection sites identified among 16 genes with synonymous or nonsynonymous mutations

**Additional file 7: Table S7.** The best substitution models in the phylogenetic analysis of *Camellia*

## Data Availability

Raw sequences data of CSSA were submitted to National Center for Biotechnology Information (NCBI) database with accession number MH042531. Other genomic data mentioned in the article can be accessed from NCBI and the details of accession number has been provided in Table 2.

## Abbreviations

BEB: Bayesian Empirical Bayes
BI: The Bayesian inference
BRIG: Blast Ring Image Generator
CAZ: *Camellia azalea*
CCR: *Camellia crapnelliana*
CCU: *Camellia cuspidate*
CDS: Protein-coding regions
CGR: *Camellia grandibracteata*
CIM: *Camellia impressinervis*
cp: Chloroplast
CPE: *Camellia petelotii*
CPI: *Camellia pitardii*
CPU: *Camellia pubicosta*
CRE: *Camellia reticulate*
CSA: *Camellia sinensis* var. assamica
CSSA: *Camellia sinensis* var. sinensis cv. Anhua
CSSL: *Camellia sinensis* var. sinensis cv. Longjing43
CSP: *Camellia sinensis var. pubilimba*
CSS: *Camellia sinensis* var. sinensis
CTA: *Camellia taliensis*
CYU: *Camellia yunnanensis*
IGS: Intergeneric regions
Indel: Insertion/deletion
IR: Inverted repeat
Ka: Nonsynonymous nucleotide substitution
Ks: Synonymous nucleotide substitution
LSC: Large single copy region
ML: The maximum likelihood
MP: The maximum parsimony
NCBI: National Center for Biotechnology Information
NJ: The neighbor-joining
PBR1: Photosystem biogenesis regulator 1
PGA: Plastid Genome Annotator
Pi: Nucleotide diversity
SNP: Single nucleotide polymorphism
SSC: Small single copy region
